# Non-targeted metabolomics unravels a media-dependent prodiginines production pathway in *Streptomyces coelicolor* A3(2)

**DOI:** 10.1371/journal.pone.0207541

**Published:** 2018-11-28

**Authors:** Yonghwan Lim, Eun Sung Jung, Je Hyeon Lee, Eun Joo Kim, Sun Joo Hong, Yeon Hee Lee, Choong Hwan Lee

**Affiliations:** 1 Department of Bioscience and Biotechnology, Konkuk University, Seoul, Korea; 2 Department of Systems Biotechnology, Konkuk University, Seoul, Korea; 3 Dynebio Incorporation, Gyeonggi-Do, Korea; Korea University, REPUBLIC OF KOREA

## Abstract

The genus *Streptomyces* is the best-known source of therapeutic secondary metabolites, especially antibiotics with pharmaceutical applications. Here, we performed a comparative study based on the time-resolved metabolic disparity in *S*. *coelicolor* A3(2) subjected to fermentative cultivation in two different types of media (R2YE and RSM3) in order to investigate secondary metabolite production pathways. The relative abundance of secondary metabolites, such as prodiginines, indoles, germicidins, and selected diketopiperazines, was increased in *S*. *coelicolor* A3(2) cultivated in R2YE medium compared to that in RSM3 medium, variably at the late-log and stationary phases of fermentative growth. Correlation analysis indicated that “antibiotic prodiginines” contributed maximally to the absorption maxima (A_530_) of culture supernatants, indicating their optimal production at 96 hours in R2YE medium. A higher abundance of L-proline (48–72 hours) followed by prodiginines (96 hours) was evident, substantiating the intertwined links between precursor and activated prodiginines pathway. Similarly, the higher abundance of indoles was concurrent with tryptophan levels in the shikimate pathway, whereas diketopiperazines were synchronously abundant along with the levels of phenylalanine, leucine, and proline. Additionally, acetyl-CoA induced the acetate pathway, resulting in the production of germicidins. Thus, our results demonstrate that *S*. *coelicolor* A3(2) produces specific secondary metabolites by enhancing the dedicated metabolic pathway responsible for their production. In conclusion, our results from this study provide insight into the metabolic pathways of *S*. *coelicolor* A3(2), and can be applied to further optimize the production of prodiginines.

## Introduction

Bacteria belonging to the genus *Streptomyces* are soil-dwelling, Gram-positive microorganisms that produce diverse bioactive compounds with commercial value. Actinobacteria, which mainly comprise the genus *Streptomyces*, produce approximately 75% of all reported bioactive compounds [1]; however, 90% of commonly used antibiotics are derived from actinomycetes [2]. *Streptomyces coelicolor* A3(2) produces distinct pigmented antibiotics, such as prodiginines (red; non-ribosomal peptide synthase/polyketide synthase-derived), actinorhodins (blue; type II polyketide), and coelimycin P1 (yellow; type I polyketide) [3]. Prodiginines have received attention not only because of their diverse pharmaceutical applications, including their use as anticancer, immunosuppressant, anti-diabetic, antibiotic, and antimalarial drugs, but also because of their commercial application, including usage in dye (textile, candles, paper, and ink production) [4]. Hence, attempts to optimize the fermentation process, medium composition, and metabolic engineering have been made [5], and a continuous effort is necessary to reduce the cost of the production of prodiginines.

Production of these bioactive compounds depends greatly on culture conditions, such as nutrient availability (carbon, nitrogen, and phosphate), incubation period, pH, and temperature in microbes [6]. Particularly, nutrient (primary metabolites) limitation strongly induces secondary metabolism in *Streptomyces* [7]; secondary metabolite production and their concentrations are also linked to primary metabolism (as precursors, cofactors, and ATP). In many cases, the selection of a specific media type is dependent on the intended purpose, such as the production of secondary metabolites, production of target proteins (enzymes), genetic manipulation, etc. The R2YE medium was specifically developed for the growth of *S*. *coelicolor* A3(2) for the production of secondary metabolites as well as for genetic manipulations [8, 9]. On the contrary, RSM3 medium was used in the past to increase the β-agarase yield, suppressing the production of secondary metabolites in the complex [10]. Since the complete genomic sequence of several *Streptomyces* species (e.g., *S*. *coelicolor* A3(2), *S*. *avermitilis*, etc.) became available, many genetic engineering studies in *Streptomyces* were actively conducted in order to enhance single bioactive compounds [3]. However, a more comprehensive study of these metabolites, such as that on the relation between primary and secondary metabolites and the transition pattern of whole metabolites in microbes, is warranted, as this information is not yet known.

Metabolomics is an analysis tool that allows qualitative and quantitative detection of all low-abundance molecular metabolites in cells and their environment at any given time. Moreover, time-course metabolite profiling provides a “static snapshot,” which allows integration of biological mechanisms and provides physiological insight [11]. Hence, metabolomic analysis coupled with system biology and metabolic engineering has the potential to further improve the metabolite production in microbes. In the past decade or so, metabolic network analyses, such as metabolic pathways analysis or metabolic flux model systems, have been applied to optimize the production of a specific substance in microbes. In *Streptomyces tenebrarius*, metabolic network analysis facilitated the purification of tobramycin from kanamycin, which differ only by one hydroxyl group. A three-times-greater reduction of the kanamycin portion of the antibiotic complex was achieved when a glucose-glycerol mixture was added as a carbon source compared to that when glucose was used alone [12]. Moreover, the metabolic flux model system revealed that the production of calcium-dependent antibiotics was dependent on nitrogen assimilation, the pentose phosphate pathway, shikimate biosynthesis, and oxoglutarate fluxes in *S*. *coelicolor* [13]. These results are applicable since they indicate the targets and strategies for genetic and process engineering manipulation for optimal production. However, to the best of our knowledge, there is a lack of research regarding analysis of secondary metabolite production pathways, including various antibiotics, particularly the pigmented antibiotic in *S*. *coelicolor* A3(2), based on a comparative metabolomics approach.

Thus, we used two different types of cultivation media (R2YE and RSM3) for the comparative study of the production of secondary metabolites in *S*. *coelicolor* A3(2) based on time-resolved untargeted metabolite profiling using gas chromatography time-of-flight-mass spectrometry (GC-TOF-MS) and ultra-performance-liquid chromatography-quadrupole (UPLC-Q)-TOF-MS. In this study, we aimed to provide information regarding secondary metabolites produced by *S*. *coelicolor* A3(2) when cultivated in two different types of media. Further, we analyzed the production of specific secondary metabolites, including antibiotics and prodiginines, by metabolic pathway analysis.

## Materials and methods

### Chemicals and reagents

All chemicals and reagents used in this study were of analytical grade. Acetonitrile, ethyl acetate, methanol, and water were purchased from Thermo Fisher Scientific (Pittsburgh, PA, USA). Formic acid, methoxyamine hydrochloride, *N*-methyl-*N*-(trimethylsilyl)-trifluoroacetamide (MSTFA), and pyridine were obtained from Sigma-Aldrich (St. Louis, MO, USA).

### *S*. *coelicolor* strain, medium composition, and culture conditions

The *Streptomyces* strain used in our study was wild type *S*. *coelicolor* A3(2), and samples of culture supernatant in R2YE and RSM3 media were provided by Dynebio Inc., (Gyeonggi-do, Korea). *S*. *coelicolor* A3(2) was maintained on MM agar containing 0.05% L-asparagine, 0.05% K_2_HPO_4_, 0.02% MgSO_4_, 0.001% FeSO_4_, and 1.5% agar in 1 L of distilled water. The strains were then cultured in a shaker at 250 rpm at 30°C in RSM3 (15 g galactose, 11 g yeast extract, 5 g MgCl_2_, and 3 g agar in 1 L of distilled water) or R2YE broth (10 g glucose, 0.25 g K_2_SO_4_, 10.12 g MgCl_2_, 0.1 g casamino acid, 5 g yeast extract, 10 mL of 0.5% KH_2_PO_4_, 80 mL of 3.68% CaCl_2_, 15 mL of 20% L-proline, 100 mL of 5.73% TES buffer [pH 7.2], and 2 mL of trace elements solution in 1 L distilled water) in order to collect their metabolic products. Detailed information regarding medium composition for the cultivation of *S*. *coelicolor* A3(2) is presented in S1 Table. The cultures were sampled at 48, 72, 96, 120, 144, and 168 hours. The sample of each culture at each time point was then centrifuged in order to compare the extracellular metabolites in the different culture conditions. Three biological replicates were performed.

### Sample preparation and metabolite profiling

#### Sample preparation and GC-TOF-MS analysis

The culture supernatants (3 mL) of *S*. *coelicolor* A3(2) in R2YE or RSM3 media were mixed with an equal volume of methanol and subjected to sonication and agitation (200 rpm for 20 minutes at 28°C) in an incubator. The sample-solvent mixtures were centrifuged (at 5000 rpm for 10 minutes at 4°C), and the solvent layers were then collected and dried using a speed vacuum concentrator (Hanil Scientific, Seoul, Korea). The weights of the dried solvent layers were then determined in order to proceed with GC-TOF-MS analysis. A derivatization step was performed for GC-TOF-MS analysis. For oximation, 50 μL of methoxyamine hydrochloride (20 mg/mL in pyridine) was added to the dried extracts and heated for 90 minutes at 30°C. Silylation was then conducted by adding 50 μL MSTFA to the mixture followed by heating at 37°C for 30 minutes. Each sample was filtered through a Millex GP 0.22 μm filter (Merck Millipore, Billerica, MA, USA). The final concentration of the derivatized samples was set at 10,000 ppm (10 mg/mL). For analytical quality, we added methyl nonadecanoate to each sample as an internal standard up to 0.25 mg/mL. The quality control (QC) sample was prepared by pooling a 10 μL aliquot from each sample. GC-TOF-MS analysis was performed using an Agilent 7890 gas chromatograph system (Agilent Technologies, Palo Alto, CA, USA) with an Agilent 7693 autosampler (Agilent Technologies) and a Pegasus HT TOF-MS (Leco Corporation, St. Joseph, MI, USA). Analytical conditions were used based on settings previously described [14]. We analyzed samples in random order to reduce bias. One QC sample was analyzed per 10 samples. All samples included three biological replicates.

#### Sample preparation and UPLC-Q-TOF-MS analysis

The culture supernatants (6 mL) of *S*. *coelicolor* A3(2) in R2YE or RSM3 media were subjected to solvent partition with an equal volume of ethyl acetate. The sample-solvent mixture was sonicated and agitated (200 rpm for 20 minutes at 28°C) in an incubator. After centrifugation (5000 rpm for 10 minutes at 4°C), the ethyl acetate layer was collected and filtered through a Millex GP 0.22 μm filter. The ethyl acetate layer was concentrated with a speed vacuum concentrator. Concentrated extracts were re-dissolved with methanol to a final concentration of 5,000 ppm (5 mg/mL). For analytical quality, we added adenosine 5′-monophosphate monohydrate, up to 0.25 mg/mL, to each sample as an internal standard. The QC sample was prepared by pooling a 10 μL aliquot from each sample. UPLC-Q-TOF-MS analysis was performed using a Waters Micromass Q-TOF Premier coupled with a UPLC Acquity system (Waters Corp., Milford, MA, USA), a binary solvent delivery system, an autosampler, and a UV detector. Analytical conditions were used based on settings previously described [15]. Samples were analyzed in random order to reduce bias, and one QC sample was analyzed per 10 samples. All samples included three biological replicates.

### Data processing and multivariate statistical analysis

**Fig 1:**
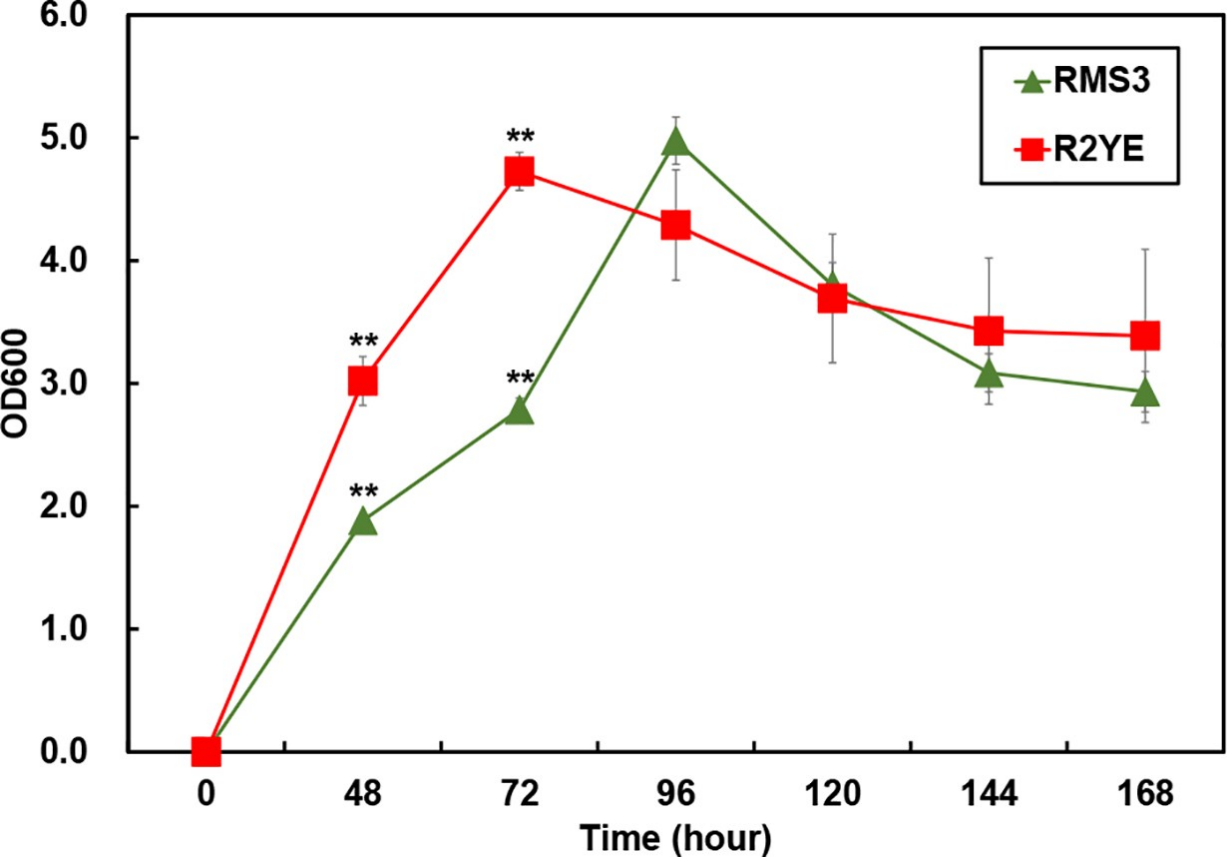
Growth curve of *S*. *coelicolor* A3(2) in R2YE and RSM3 media. The absorbance of culture supernatants was measured at 600 nm. (Red square, *S*. *coelicolor* A3(2) in R2YE medium; green triangle, *S*. *coelicolor* A3(2) in RSM3 medium). ** Significant differences calculated by student t-test (*P* < 0.001).

The raw data sets of GC-TOF-MS were converted to the Net CDF format (*.cdf) using the LECO Chroma TOF software (version 4.44, LECO Corpo). The UPLC-Q-TOF-MS data sets were converted to the Net CDF format using MassLynx DataBridge (version 4.1, USA, Waters Corp.). Converted CDF data were preprocessed with the MetAlign software package (http://www.metalign.nl) for peak detection, retention time correction, and alignment. The resulting data, including sample names and peak area information as variables, were exported to an Excel file. Data was normalized to the growth rate of *S*. *coelicolor* A3(2) in R2YE and RSM3 media (Fig 1). Data sets then were scaled to internal standards based on peak intensities of methyl nonadecanoate (for GC-TOF-MS) and adenosine 5′-monophosphate monohydrate (for UPLC-Q-TOF-MS) for analytical quality of the results. Multivariate statistical analysis was conducted using SIMCA-P+ (version 12.0, Umetrics, Umea, Sweden). The data sets were subjected to auto-scale and mean-center in a column-wise manner. Then principal component analysis (PCA), partial least squares discriminant analysis (PLS-DA), and orthogonal PLS-DA (OPLS-DA) modeling were performed to compare each data set. The detected metabolites were tentatively identified by comparison with mass fragment patterns, retention time, and mass spectrum of analysis data for standard compounds under the same conditions described in literature and commercial databases, such as the National Institutes of Standards and Technology (NIST) Library (version 2.0, 2011, FairCom, Gaithersburg, MD, USA), The Dictionary of Natural Products (version 16:2, 2007, Chapman & Hall, USA), Wiley 8, BioCyc Database Collection (https://biocyc.org/), and the Human Metabolome Database (HMDB; http://www.hmdb.ca/). Significant differences (*P* < 0.05) were tested by one-way ANOVA using Statistica (version 7.0, StatSoft Inc., Tulsa, OK, USA). A heat map was constructed using MultiExperiment Viewer software (MeV) software (http://www.tm4.org/) to compare the disparity of metabolites. A correlation map was obtained using PASW Statistics 18.0 software (SPSS Inc., Chicago, IL, USA).

### UV-visible absorption measurement

Absorbance variations of *S*. *coelicolor* A3(2) culture supernatants in R2YE or RSM3 media were measured using a Cary 100 UV-Vis Spectrophotometer (Agilent Technologies, Palo Alto, CA, USA) from 350–700 nm in a time-dependent manner (0, 48, 72, 96, 120, 144, and 168 hours). Scan controls were as follows: average time 0.1 s, data interval 1.0 nm, and scan rate 600.0 nm/min. A correlation assay for the absorbance of each culture supernatant and secondary metabolites in *S*. *coelicolor* A3(2) was calculated specifically for actinorhodin (blue-pigment, A_640_), prodiginine (red-pigment, A_530_), and coelimycin P1 (yellow-pigment, A_450_). Corrected readings were performed to eliminate the influence of actinorhodin: corrected A_450_ = A_450_ − 0.86 × A_640_ [16].

## Results

### Media-dependent differences in secondary metabolite production based on the cultivation media of *S*. *coelicolor* A3(2)

**Fig 2:**
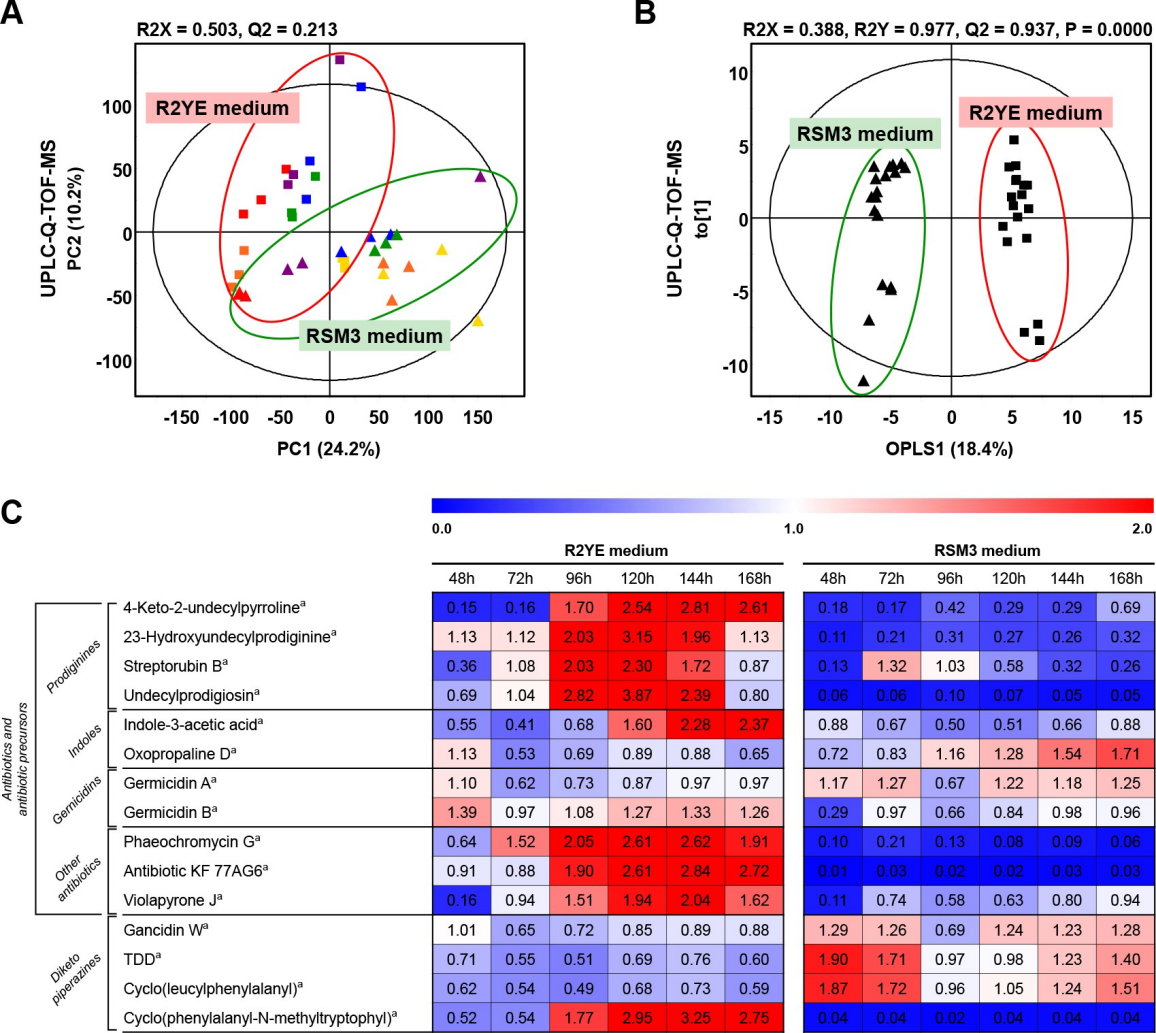
**PCA score plot (A), OPLS-DA score plot (B), and heat map representations (C) of secondary metabolites produced by *S*. *coelicolor* A3(2) grown in R2YE and RSM3 media based on the UPLC-Q-TOF-MS data set.** Data were normalized to the growth rates (Fig 1) of *S*. *coelicolor* A3(2) in R2YE and RSM3 media and then were scaled to an internal standard using adenosine 5′-monophosphate monohydrate. Significantly different metabolites in the heat map were selected by VIP values (> 0.7). Each column represents fold change normalized to the average of all values and shows blue (0) to red (2). (Square, *S*. *coelicolor* A3(2) in R2YE medium; triangle, *S*. *coelicolor* A3(2) in RSM3 medium; yellow, 48 h; orange, 72 h; red, 96 h; purple, 120 h; blue, 144 h; green, 168 h).^a^ It was selected by *p*-value (< 0.05). TDD: Tryptophan-dehydrobutyrine diketopiperazine.

The differences in time-resolved secondary metabolite production based on *S*. *coelicolor* A3(2) cultivation in two different media (R2YE and RSM3) were examined by MS-based metabolite profiling combined with multivariate statistical analysis. The analytical quality of UPLC-Q-TOF-MS was verified by the QC samples, which showed a grouping pattern in PCA score plots (S1B Fig). As shown in Fig 2A and 2B, PCA and OPLS-DA score plots based on UPLC-Q-TOF-MS data profiling demonstrated distinct separating patterns between R2YE and RSM3 along PC1 (24.2%) and OPLS1 (18.4%) components with corresponding statistically significant model values of R^2^X, R^2^Y, and Q^2^, as shown in the OPLS-DA score plot. In the PCA score plot, the secondary metabolites of *S*. *coelicolor* A3(2) produced in two different types of media (R2YE and RSM3) changed consistently in a time-dependent manner (48, 72, 96, 120, 144, and 168 hours), with separating patterns between the two types of media. Based on the OPLS-DA score plot (Fig 2B), a total of 15 significantly different secondary metabolites produced by *S*. *coelicolor* A3(2) grown in R2YE and RSM3 media were selected based on variable importance projection (VIP) values (> 0.7) and *p*-values (< 0.05). Detailed information of the metabolites that were significantly different between R2YE and RSM3, including four prodiginines, four diketopiperazines, two indoles, two germicidins, and three other antibiotics, is presented in S4 Table. The relative production levels of the secondary metabolites produced by *S*. *coelicolor* A3(2) in the two different types of media in a time-dependent manner are shown as a heat map (Fig 2C). Media-specific secondary metabolites were produced by *S*. *coelicolor* A3(2). At 96 hours, we observed a significantly increased relative abundance of prodiginines (4-keto-2-undecylpyrroline, 23-hydroxyundecylprodiginine, streptorubin B, and undecylprodigiosin), several antibiotics (indole-3-acetic acid, germicidin B, phaeochromycin G, antibiotic KF 77AG6, and violapyrone J), and cyclo(phenylalanyl-N-methyltryptophyl) in *S*. *coelicolor* A3(2) cultivated in R2YE medium compared to RSM3 on the basis of VIP(> 0.7) and *p*-value (< 0.05). In contrast, at 120 hours we observed an increase in the relative content of most diketopiperazines (gancidin W, tryptophandehydrobutyrine diketopiperazine (TDD), and cyclo(leucylphenylalanyl)) and several antibiotics (oxopropaline D and germicidin A) produced by *S*. *coelicolor* A3(2) in RSM3 medium compared to R2YE medium.

### Correlation between the time-resolved UV-visible absorption variation and pigmented antibiotic production by *S*. *coelicolor* A3(2) in R2YE media

**Fig 3:**
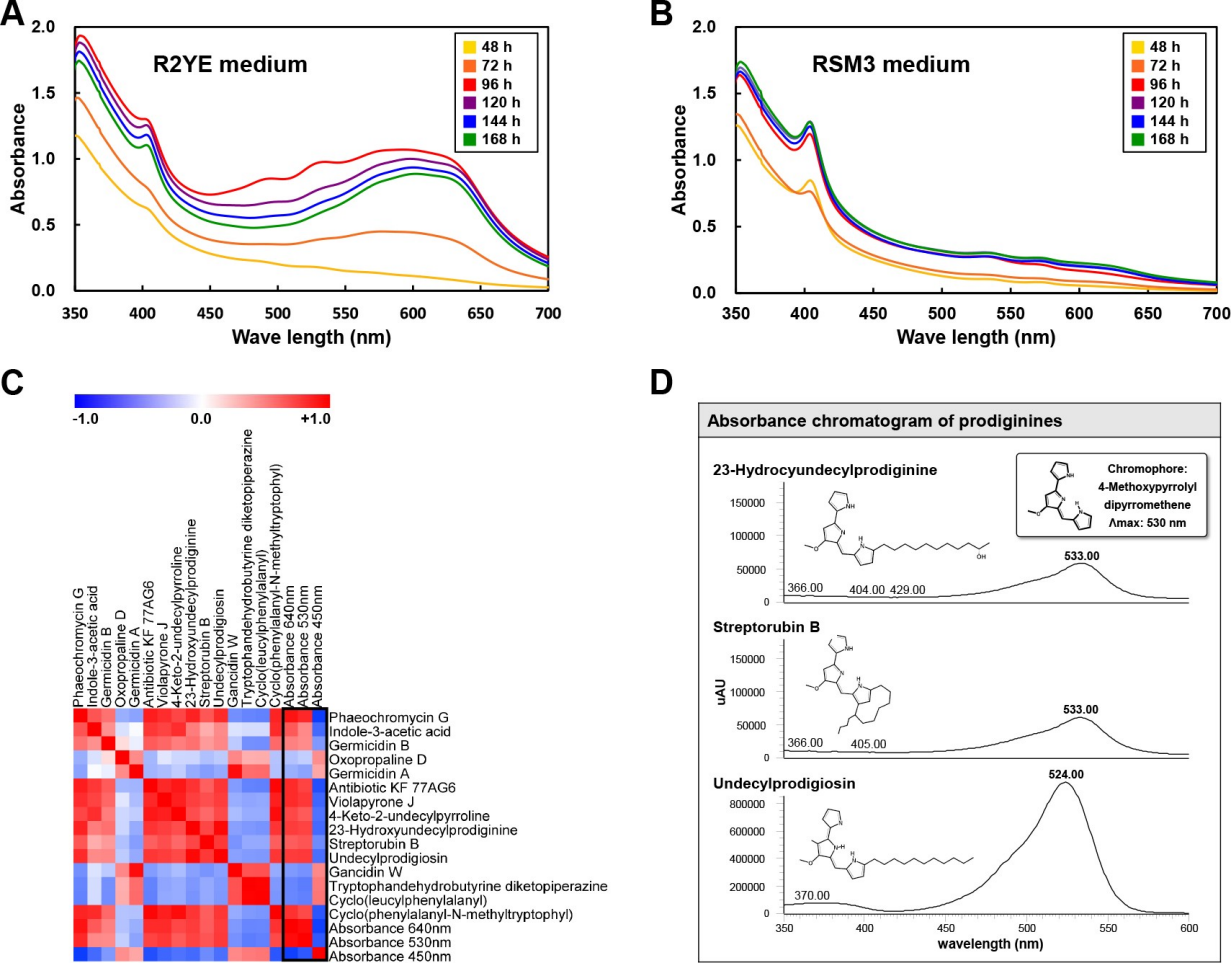
**Absorbance variation in culture supernatants of *S*. *coelicolor* A3(2) in R2YE (A) and RSM3 (B) media at different time points, a correlation map between the detected secondary metabolites and absorbance measurements for A**_**640**_**, A**_**530**_**, and A**_**450**_ **(C), and the absorbance chromatogram for prodiginines derived from UHPLC-LTQ-ESI-IT-MS/MS coupled with diode array detector (D).** Each square in the correlation map represents Pearson's correlation coefficient values (r), blue color indicates negative correlation (−1 < r < 0), and red color indicates positive correlation (0 < r < 1). (yellow, 48 h; orange, 72 h; red, 96 h; purple, 120 h; blue, 144 h; green, 168 h).

Different types of media resulted in different growth rates of *S*. *coelicolor* A3(2) (Fig 1). While *S*. *coelicolor* A3(2) reached maximum growth at 72 hours in R2YE medium, *S*. *coelicolor* A3(2) did not reach maximum growth in RSM3 medium until 96 hours. With R2YE medium, pigment compounds were released during growth, which lead to a change in the color of the medium based on *S*. *coelicolor* A3(2) growth. We measured the absorbance (350–700 nm) of *S*. *coelicolor* A3(2) culture supernatants in each media over a time course (48, 72, 96, 120, 144, and 168 hours) (Fig 3A and 3B). Absorbance measurements showed variation for each media at different growth times. The overall absorbance of *S*. *coelicolor* A3(2) growth in R2YE medium increased substantially until 96 hours, at which time it gradually decreased, whereas the absorbance of *S*. *coelicolor* A3(2) growth in RSM3 medium was unchanged during the time course. To determine the correlation between the absorbance of culture supernatant and secondary metabolite production, correlation analyses were performed (Fig 3C). According to the correlation map, elevated associations of secondary metabolites, including 4-keto-2-undecylpyrroline, 23-hydroxyundecylprodiginine, streptorubin B, undecylprodigiosin, phaeochromycin G, indole-3-acetic acid, antibiotic KF 77AG6, violapyrone J, and cyclo(phenylalanyl-N-methyltryptophyl) with A_640_ and A_530_ were observed. Among them, 4-keto-2-undecylpyrroline, 23-hydroxyundecylprodiginine, streptorubin B, and undecylprodigiosin were prodiginine compounds. In the diode array detector (DAD) data, we also found that undecylprodigiosin, streptorubin B, and 23-hydroxyundecylprodiginine demonstrated absorbance at 530 nm (Fig 3D). These compounds commonly include the chromophore structure of 4-methoxypyrrolyl-dipyrromethene.

### Media-dependent secondary metabolite production pathway of *S*. *coelicolor* A3(2) based on metabolite profiling

**Fig 4:**
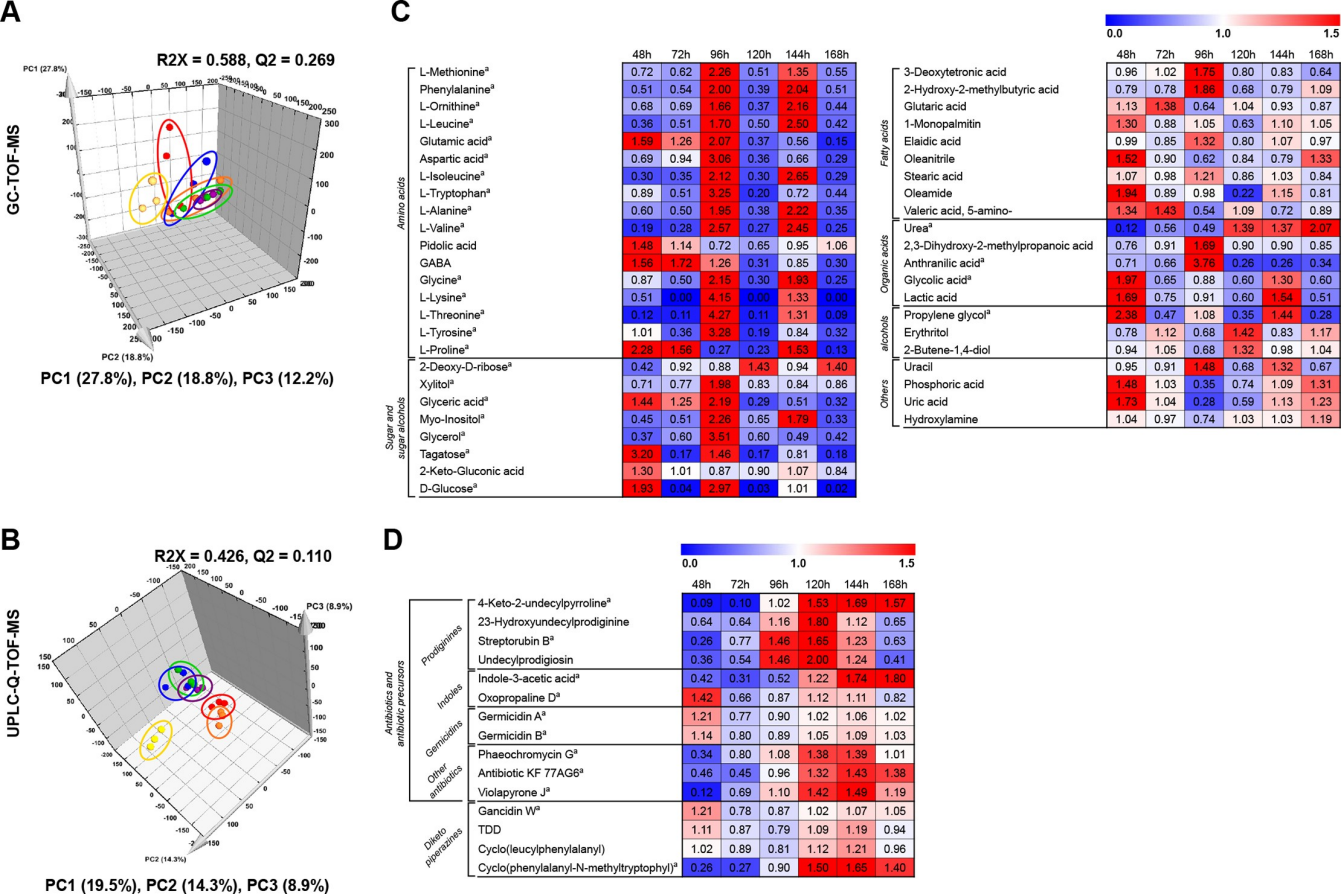
**PCA score plots of primary (A) and secondary metabolites (B) and heat map representations of changes in the relative content of significantly discriminant primary metabolites (C) and secondary metabolites (D) in *S*. *coelicolor* A3(2) in R2YE medium.** Primary and secondary metabolite data derived from GC-TOF-MS and UPLC-Q-TOF-MS, respectively. Data were normalized to the growth rates (Fig 1) of *S*. *coelicolor* A3(2) in R2YE medium and were scaled to internal standards using methyl nonadecanoate (for GC-TOF-MS analysis) and adenosine 5′-monophosphate monohydrate (for UPLC-Q-TOF-MS). Significantly discriminant metabolites in the heat map were selected by VIP values (> 0.7) and each column represents the fold change normalized to an average of all values and shows blue (0) to red (1.5). (yellow, 48 h; orange, 72 h; red, 96 h; purple, 120 h; blue, 144 h; green, 168 h). ^a^ It was selected by *p*-value (*P* < 0.05)..TDD: Tryptophandehydrobutyrine diketopiperazine.

**Fig 5:**
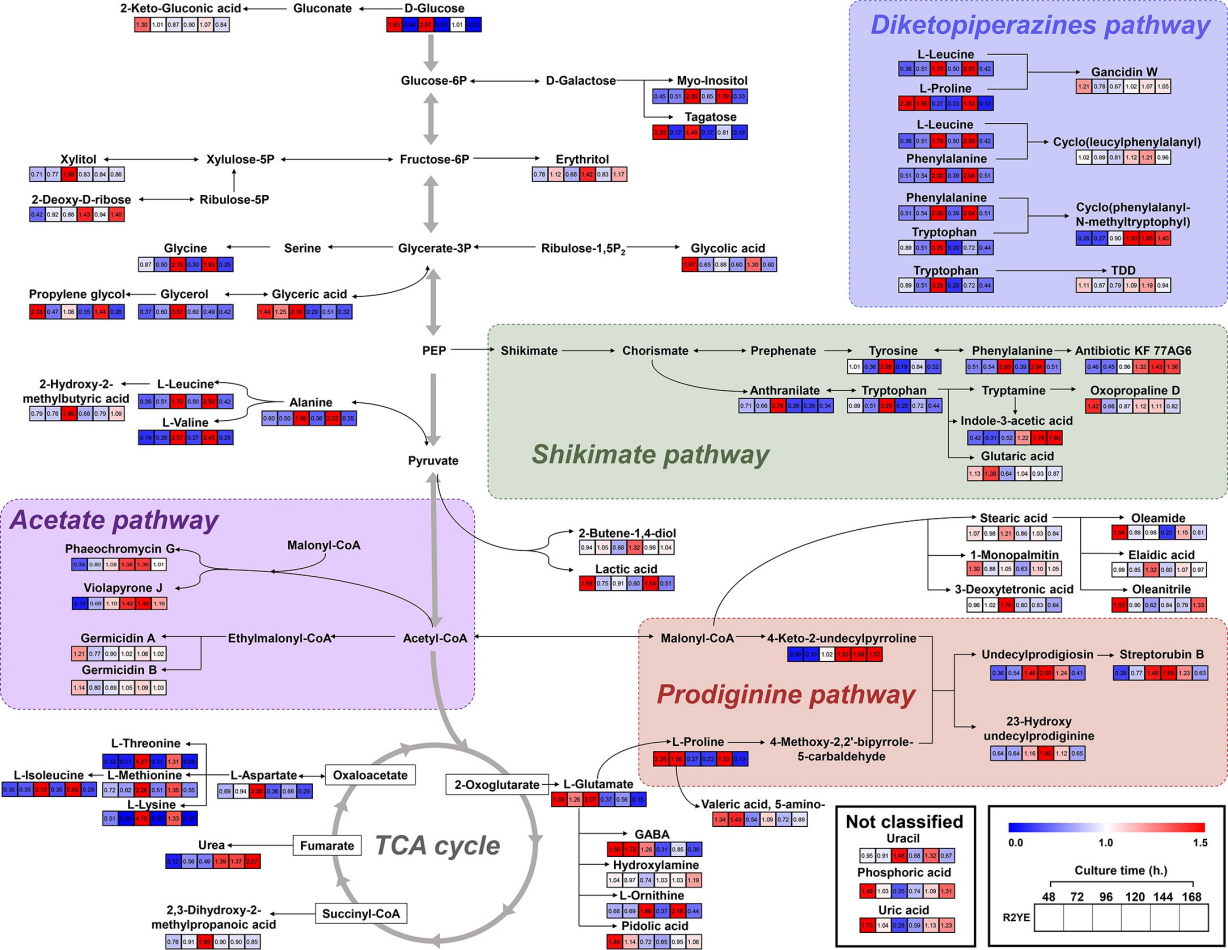
The metabolic pathway and relative contents of *S*. *coelicolor* A3(2) metabolites produced in R2YE medium at different time points. Primary and secondary metabolite data derived from GC-TOF-MS and UPLC-Q-TOF-MS, respectively. Data were normalized based on the growth rates (Fig 1) of *S*. *coelicolor* A3(2) in R2YE medium and were scaled to internal standards using methyl nonadecanoate (for GC-TOF-MS analysis) and adenosine 5′-monophosphate monohydrate (for UPLC-Q-TOF-MS). Metabolite data was selected by VIP values (> 0.7). The pathway map was modified from the Kyoto Encyclopedia of Genes and Genomes (KEGG) (http://www.genome.jp/kegg/). Fold change was normalized to an average of all values and is shown as blue (0) to red (2).

To investigate the secondary metabolite production pathway, we focused on *S*. *coelicolor* A3(2) growth in R2YE medium, which produced more secondary metabolites. Time-resolved untargeted metabolome profiling analyzed by GC-TOF-MS was performed. Analytical quality of GC-TOF-MS was verified by QC samples, which showed a grouping pattern in the PCA score plot (S1A Fig). The PCA score plot derived from GC-TOF-MS datasets exhibited obvious grouping patterns of the metabolites produced by *S*. *coelicolor* A3(2) grown in R2YE media based on the time point: 48 hours; 72, 120, and 168 hours; 96 hours; and 144 hours with PC1 (27.8%), PC2 (18.8%), and PC3 (12.2%) (Fig 4A). In addition, time-resolved untargeted metabolome profiling with UPLC-Q-TOF-MS was conducted and the analytical quality was verified (S1B Fig). The PCA score plot derived from UPLC-Q-TOF-MS data sets also exhibited time-dependent grouping patterns: 48 hours; 72 hours; 96 hours; and 120, 144, 168 hours with PC1 (19.5%), PC2 (14.3%), and PC3 (8.9%) (Fig 4B). For comparative studies, we performed data processing with PLS-DA models (S1C and S1D Fig) based on the PCA grouping patterns of primary and secondary metabolites of *S*. *coelicolor* A3(2) in R2YE medium. In total, 61 metabolites were putatively identified as significantly discriminant metabolites in accordance with the VIP values (> 0.7) based on grouping patterns (S2 and S3 Tables). These compounds included 46 primary metabolites (17 amino acids, 9 fatty acids, 8 sugars, 5 organic acids, 3 alcohols, and 4 others) and 15 secondary metabolites (4 prodiginines, 4 diketopiperazines, 2 indoles, 2 germicidins, and 3 other antibiotics). The relative contents of primary and secondary metabolites at different time points of *S*. *coelicolor* A3(2) growth in R2YE medium are represented in Fig 4C and 4D, respectively. We observed a relatively high abundance of most amino acids and sugars at 96 hours. Also at 96 hours, production of most of the secondary metabolites was initiated by *S*. *coelicolor* A3(2), which then increased until 120–144 hours. After that, the levels of secondary metabolites decreased. Using these metabolites, we constructed a metabolic pathway (Fig 5). In this metabolic pathway, secondary metabolites, including antibiotics and diketopiperazines, were primarily produced by four different pathways. The pigmented antibiotic prodiginine was produced from the precursor of L-proline. A higher abundance of L-proline at 48–72 hours followed by prodiginine production at 96 hour was observed. From this pathway, prodiginines, such as undecylprodigiosin, streptorubin B, and 23-hydroxyundecylprodiginine, were produced. Prodiginines were formed by the condensation of precursors derived from L-proline, and 4-keto-2-undecylpyrroline was derived from malonyl-CoA. By the shikimate pathway, shikimate-derived compounds, tryptophan and phenylalanine (96 hours), were converted into indoles (oxopropaline D and indole-3-acetic acid) and antibiotic KF77AG6, respectively. Similarly, a higher abundance of several amino acids at 96 hours, including L-leucine, L-proline, phenylalanine, and tryptophan, contributed to the production of diketopiperazines (gancidin W, cyclo(leucylphenylalanyl), cyclo(phenylalanyl-N-methyltryptophyl), and tryptophandehydrobutyrine diketopiperazine) at 120 hours by *S*. *coelicolor* A3(2) in R2YE medium. Lastly, several antibiotics, such as germicidins (germicidin A and germicidin B), phaeochromycin G, and violapyrone J, were produced from acetyl-CoA- and malonyl-CoA-derived precursors at 72 hours.

## Discussion

In *Streptomyces*, secondary metabolism is generally confined to the stationary phase and is thought to result from nutrient limitation or a reduction in growth rate, which is the signal serving as the “trigger” for secondary metabolism [7]. Through the comparative study of the secondary metabolites produced by *S*. *coelicolor* A3(2) when cultivated in R2YE and RSM3 media, we revealed that the nutrient-rich R2YE medium induced specific production of secondary metabolites, especially prodignine, while RSM3 media did not induce the production of specific secondary metabolites (Fig 2C). In addition, different growth rates were observed between the two types growth media, with maximum growth occurring at 72 hours and 96 hours in R2YE medium and RSM3 medium, respectively (Fig 1). The R2YE medium is a nutrient-rich medium, with a greater variety of carbons, nitrogens, and trace metals, as well as a mixture of other ingredients, such as yeast extract and casamino acids, compared to the RSM3 medium, which is nutrient-restrictive (S1 Table and S2 Fig). Previous studies have shown that different types of bacteria cultivated in complex media exhibit a faster growth rate and produce relatively higher contents of metabolic end-products compared to those cultivated in minimal medium [17]. Microorganisms take up nutrients, such as carbon and nitrogen sources, from the environment and simultaneously produce intermediates and end-products for growth [18]. However, when essential nutrients become depleted, bacterial cells enter stationary phase with decreased growth and produce secondary metabolites by reorganizing their metabolism [19]. Herein, we can assume that *S*. *coelicolor* A3(2) grew rapidly in R2YE medium by consuming the available nutrients at 72 hours, subsequently producing specific secondary metabolites, such as prodiginines (Fig 2C), relative to RSM3 medium. Unlike R2YE media, RSM3 media induced an accumulation of energy storage metabolites rather than secondary metabolites (S3 Fig). Previously, it was reported that certain bacteria in minimal media primarily synthesize sugars and fatty acids, which are essential for the biosynthesis of polysaccharides and lipids, both of which are important for energy storage and survival in nutrient-restrictive conditions [20, 21]. On the other hand, microorganisms in complex media produce a higher relative abundance of amino acids, which are known to be essential for protein synthesis and cell growth [22]. This phenomenon was partially consistent with the variation of primary metabolites in our study (S3 Fig). We observed a relatively higher abundance of primary metabolites, including sugars, alcohols, fatty acids, organic acids, alcohols, and pyrimidines with significant increases measured up to 144 hours in RSM3 medium compared to R2YE medium. Thus, the R2YE medium was actively consumed by *S*. *coelicolor* A3(2) and induced a metabolic switch to active secondary metabolism, while the RSM3 medium caused *S*. *coelicolor* A3(2) to utilize primary metabolism only for energy storage. In this study, we investigated the extracellular metabolism of *S*. *coelicolor* A3(2), however, further research regarding both the intra- and extracellular metabolism is needed in order to completely understand the entire metabolic pathways in *S*. *coelicolor* A3(2).

Based on our comprehensive metabolomics analysis, we constructed a metabolic pathway using the primary and secondary metabolites produced by *S*. *coelicolor* A3(2) in R2YE medium (Fig 5). From the pathway analysis, we revealed an association between the primary metabolism and the production of essential secondary metabolites in *S*. *coelicolor* A3(2). Secondary metabolism generally occurs through dedicated biosynthetic pathways in *S*. *coelicolor* A3(2), which are linked to the corresponding primary metabolites through the supply precursors. According to this pathway model, four major biosynthetic routes of secondary metabolite production were observed, including prodiginine, shikimate, acetate, and diketopiperazine pathway.

In the prodiginines pathway, L-proline is an essential intermediate for prodiginine biosynthesis, namely undecylprodigiosin and streptorubin B, in *S*. *coelicolor* A3(2) cultivation. In our study, we used R2YE medium, which included 0.3% L-proline for *S*. *coelicolor* A3(2) cultivation. Earlier studies suggested that the biosynthesis of prodigiosin is affected by the consumption of NAD(P)H or proline [23] through the primary metabolism in *Streptomyces*. Structurally, L-proline directly incorporates into prodigiosin biosynthesis [24], which increases the cellular pool of prodigiosin in non-proliferating cells [25]. In addition, expression of related genes, i.e., the *red* gene cluster consisting of 23 genes, is implicated in prodiginine biosynthesis in *S*. *coelicolor* through L-proline [26]. Moreover, malonyl-CoA, formed from acetyl-CoA (Embden–Meyerhof–Parnas pathway), was directly affected by the central carbon metabolism [12] and influenced the production of the red-pigmented antibiotic prodiginine (e.g., 23-hydroxyundecylprodiginine, undecylprodigiosin, and streptorubin B), by production of 4-keto-2-undecylpyrroline. Through previous studies on prodiginine biosynthesis, we know that undecylprodigiosin and streptorubin B are formed when two different biosynthetic pathway compounds (4-methoxy-2,2’-bipyrrole-5-carbaldehyde and 2-undcylpyrrole) are condensed together, which are downstream products of L-proline and malonyl-CoA, respectively [26].

In the shikimate pathway, shikimic acid is the precursor of phenylalanine and tryptophan, which are intermediates for the production of the antibiotic KF 77AG6 (phenylalanine), oxopropaline D, and indole-3-acetic acid (tryptophan). We observed the activated shikimate pathway through a relatively high abundance of tryptophan at 96 hours, followed by increasing patterns of oxopropaline D and indole-3-acetic acid at 120 hours. According to a previous report, the phytohormone indole-3-acetic acid production by *Streptomyces* is dependent upon tryptophan availability [27]. Earlier research revealed that indole-3-acetic acid production was induced 61-fold upon the exogenous addition of 10 mM tryptophan to the growth media of *Streptomyces scabiei* compared to that in the normal *S*. *scabiei* group [28]. The *iaaM* and *iaaH* genes, which encode the enzymes tryptophan mono-oxygenase (IaaM) and indole-3-acetamide hydrolase (IaaH), respectively, are known to be involved in indole-3-acetic acid production [29]. Neither *iaaM* nor *iaaH* demonstrated transcriptional induction by tryptophan, but dedicated enzymatic activities are tryptophan-dependent at various concentrations [28]. Hence, we assume that the moderately increased content of tryptophan observed at 96 hours was part of the activated shikimate pathway and contributed to the biosynthesis of indole-3-acetic acid by *S*. *coelicolor* A3(2) cultivated in R2YE medium.

Through the acetate pathway, several antibiotics, including germicidin A, germicidin B, phaeochromycin G, and violapyrone J, were produced by *S*. *coelicolor* A3(2) grown in R2YE medium. The acetyl-CoA produced by *S*. *coelicolor* A3(2) is a key intermediate for the above-mentioned compounds, which were reportedly identified from various *Streptomyces* species [30, 31, 32]. Acetyl-CoA is implicated in the central carbon metabolism of microbes and is located downstream of carbon sources and a variety of cellular processes in microbes [33]. We observed a relatively high abundance of glucose at 96 hours, which may potentially further influence the production of compounds from the acetyl-CoA and acetate pathways, including germicidin A and germicidin B., in *S*. *coelicolor* A3(2) grown in R2YE medium. Furthermore, malonyl-CoA is produced exclusively through acetyl-CoA carboxylation by acetyl-CoA carboxylase and is known to be used in the production of polyketide compounds in *S*. *coelicolor* A3(2) [34]. As shown in Fig 5, malonyl-CoA influenced the production of antibiotics in the acetate pathway, such as phaeochromycin G and violapyrone J.

Most of the diketopiperazines in our results, including gancidin W, cyclo(leucyl-phenylalanyl), cyclo(phenylalanyl-N-methyltryptophyl), and tryptophan-dehydrobutyrine, were synchronously abundant with dedicated precursors, such as L-leucine, L-proline, phenylalanine, and tryptophan (Figs 4 and 5). The basic structure of diketopiperazines, i.e., 2,5-diketopiperazines, is usually formed by the condensation of two α-amino acids. Diketopiperazines are commonly found in a variety sources, such as bacteria, fungi, plants, and mammals [35]. Diketopiperazines are regarded as attractive scaffolds for drug discovery due to their proteolytic stability, rigid backbone, ability to mimic a preferential peptide conformation, and diversity of substituent groups; furthermore, diketopiperazines do not possess the poor physical and metabolic properties of peptides [36]. However, only few studies have reported the biological activity of diketopiperazines; hence, additional studies on the bioactivity of diketopiperazines are needed.

In our results, the overall absorbance of *S*. *coelicolor* A3(2) growth in R2YE medium increased substantially up until 96 hours, at which point it gradually decreased. The growth of *S*. *coelicolor* A3(2) growth in R2YE medium correlated with the production of pigmented antibiotics, such as 4-keto-2-undecylpyrroline, 23-hydroxyundecylprodiginine, streptorubin B, and undecylprodigiosin (Fig 3). These changing patterns of absorbance are a result of the unique ability of *S*. *coelicolor* to produce pigmented antibiotics. Reportedly, these highly correlated compounds are known as a family of red-pigmented antibiotics produced by actinomycetes, including *Streptomyces* [37]. For several decades, many researchers have attempted to investigate various pigmented polyketide compounds in *S*. *coelicolor* A3(2), including red-pigmented prodiginine (*red* gene), blue-pigmented actinorhodin (*act* gene), yellow-pigmented coelimycin P1 (*cpk* gene), grey spore pigment (*whiE* gene), etc. [38]. The productivity of each pigmented antibiotic in *S*. *coelicolor* A3(2) is differently influenced by the nutrients in media, including carbon, nitrogen, and trace metals. Carbon source regulation, known as carbon catabolite repression (CCR), generally occurs with competition in the microbial environment [39]. In particular, glucose is a key regulator of this phenomenon in *Streptomyces*, and thus represses (e.g., actinorhodin) or reduces (e.g., undecylprodigiosin) production by regulating the dedicated precursors [40]. In case of our cultivation, we used R2YE medium with 1% glucose as a carbon source, which has the potential to highly influence the production of red-pigmented prodiginines (S1 Table and Fig 5). In terms of phosphate, we also observed a remarkable decrease in phosphoric acid at 96 hours (Fig 4C). In many studies, the relationship between phosphate levels and secondary metabolite production has been examined. Excessive phosphate represses the formation of diverse secondary metabolites through phosphorylated polyphosphate kinase, PhoP [7], whereas inactivation of polyphosphate kinase reportedly caused an increase in actinorhodin, prodiginine, and calcium-dependent antibiotic production in *Streptomyces lividans* [41]. According to a previous study, actinorhodin production by *S*. *coelicolor* A3(2) grown in Hepatocyte Maintenance Medium with phosphate induced complete inhibition of actinorhodin at concentrations greater than 24 mM of phosphate, whereas undecylprodgiosin was still produced at the same concentration [42]. Trace metals are intimately related to the morphological development and secondary metabolite production in *Streptomyces* as metalloregulators and cofactors of biosynthetic enzymes [43]. We used various trace elements (ZnCl_2_, FeCl_2_, CuCl_2_, MnCl_2_, Na_2_B_4_O_7_, (NH_4_)_6_Mo_7_O_24_) in R2YE medium, but not in RSM3 medium for *S*. *coelicolor* A3(2) cultivation. Iron-derived medium is reported to negatively affect actinorhodin production in *S*. *coelicolor* A3(2) [44]. Hence, we tentatively assume that one of the reasons for no detection of blue-pigmented actinorhodin in *S*. *coelicolor* A3(2) is the regulation of the production of this pigment by trace elements in R2YE medium.

In conclusion, we examined the time-resolved metabolic disparity in *S*. *coelicolor* A3(2) subjected to fermentative cultivation in two different types of media, nutrient-rich R2YE media and nutrient-restrictive RSM3 media, in order to investigate the secondary metabolite production pathways. In terms of metabolism, *S*. *coelicolor* A3(2) grown in R2YE medium underwent early primary metabolism followed by a metabolic switch to secondary metabolism, while *S*. *coelicolor* A3(2) grown in RSM3 medium remained in a state of primary metabolism for energy storage (S3 Fig). To the best of our knowledge, we report here the first study regarding metabolic pathway analysis for the production of secondary metabolites, especially prodiginines, produced by *S*. *coelicolor* A3(2) cultivated in R2YE medium. Understanding the metabolic network through metabolic pathway analysis of *S*. *coelicolor* A3(2) grown in R2YE medium is required in order to establish genetic engineering targets and process operations, including media formulation and cultivation periods, with the goal of reducing the time and cost for the optimal production of prodiginines.

## Supporting information

S1 Table Medium composition for *S*. *coelicolor* A3(2) cultivation. (DOCX)

S2 Table Secondary metabolites produced by *S*. *coelicolor* A3(2) grown in R2YE and RSM3 media tentatively identified by time-resolved cultivation analyzed by UPLC-Q-TOF-MS. ^a^ Retention time; ^b^ Metabolites selected by VIP value > 0.7 based on OPLS-DA (Fig 2B); ^c^ It was selected by *p*-value (< 0.05) based on one-way ANOVA analysis. ^d^ Identification. CCD, *The Dictionary of Natural Products* (version 16:2, 2007, Chapman & Hall, USA); BioCys, Identification of metabolites was carried out using BioCyc Database Collection (https://biocyc.org/); HMDB, Identification of metabolites was carried out using the Human Metabolome Database (HMDB; http://www.hmdb.ca/).
(DOCX)

S3 Table Primary metabolites produced by *S*. *coelicolor* A3(2) grown in R2YE medium tentatively identified by time-resolved cultivation analyzed by GC-TOF-MS. ^a^ Retention time; ^b^ Metabolites selected by VIP value > 0.7 based on PLS-DA (S1C Fig); ^c^ It was selected by *p*-value (< 0.05) based on one-way ANOVA analysis; ^d^ Identification. MS, mass spectrum was confirmed with the National Institutes of Standards and Technology (NIST) database and in-house libraries; STD, mass spectrum was consistent with that of the standard compounds; TMS, trimethylsilyl.
(DOCX)

S4 Table Secondary metabolites produced by *S*. *coelicolor* A3(2) grown in R2YE medium tentatively identified by time-resolved cultivation analyzed by UPLC-Q-TOF-MS. ^a^ Retention time; ^b^ Metabolites selected by VIP value > 0.7 based on PLS-DA (S1D Fig); ^c^ It was selected by *p*-value (< 0.05) based on one-way ANOVA analysis. ^d^ Identification. CCD, *The Dictionary of Natural Products* (version 16:2, 2007, Chapman & Hall, USA); BioCys, Identification of metabolites was carried out using BioCyc Database Collection (https://biocyc.org/); HMDB, Identification of metabolites was carried out using the Human Metabolome Database (HMDB; http://www.hmdb.ca/).
(DOCX)

S1 Fig **PCA score plots with QC of primary (A) and secondary metabolites (B) produced by *S. coelicolor* A3(2) grown in R2YE and RSM3 media, and PLS-DA score plots of primary metabolites (C) and secondary metabolites (D) produced by *S. coelicolor* A3(2) grown in R2YE medium**. The analysis was performed with GC-TOF-MS and UPLC-Q-TOF-MS. Data were scaled to internal standards using methyl nonadecanoate (for GC-TOF-MS analysis) and adenosine 5′-monophosphate monohydrate (for UPLC-Q-TOF-MS analysis). (Star, quality control; square, *S. coelicolor* A3(2) in R2YE medium; triangle, *S. coelicolor* A3(2) in RSM3; yellow, 48 h; orange, 72 h; red, 96 h; purple, 120 h; blue, 144 h; green, 168 h).
(DOCX)

S2 Fig **Metabolites in R2YE (A) and RSM3 (B) analyzed by GC-TOF-MS.** Metabolites were tentatively identified by comparison with mass fragment patterns, retention time, and mass spectrum of analysis data for standard compounds under the same conditions and commercial databases, such as the NIST Library and Wiley 8. Metabolite numbers are as follows: 1. L-alanine, 2. L-valine, 3. L-leucine, 4. L-isoleucine, 5. L-proline, 6. glycine, 7. serine, 8. L-threonine, 9. aspartic acid, 10. L-methionine, 11. pidolic acid, 12. GABA, 13. glutamic acid, 14. phenylalanine, 15. L-ornithine, 16. L-lysine, 17. L-tyrosine, 18. L-tryptophan, 19. glycerol, 20. glyceric acid, 21. threonic acid, 22. 2-deoxy-D-ribose, 23. 2-keto-gluconic acid, 24. xylose, 25. arabinose, 26. xylitol, 27. tagatose, 28. D-galactose, 29. D-glucose, 30. D-gluconic acid, 31. myo-inositol, 32. 2-hydroxy-2-methylbutyric acid, 33. 4-hydroxybutanoic acid, 34. glutaric acid, 35. 3-deoxytetronic acid, 36. valeric acid, 5-amino-, 37. oleanitrile, 38. elaidic acid, 39. stearic acid, 40. oleamide, 41. 1-monopalmitin, 42. lactic acid, 43. glycolic acid, 44. urea, 45. benzoic acid, 46. 2,3-dihydroxy-2-methylpropanoic acid, 47. anthranilic acid, 48. propylene glycol, 49. 2,3-butanediol, 50. 2-butene-1,4-diol, 51. carbitol, 52. erythritol, 53. uracil, 54. cytosine, 55. 2-pyrrolidinone, 56. hydroxylamine, 57. phosphoric acid, and 58. uric acid.
(DOCX)

S3 Fig Heat map representations of the relative content of significantly discriminant primary metabolites produced by *S*. *coelicolor* A3(2) cultivated in R2YE and RSM3 media analyzed by GC-TOF-MS. Significantly discriminant metabolites were selected by VIP values > 0.7. Fold change was normalized to an average of all values and is shown as blue (0.0) to red (2.0). ^a^ Selection by *p*-value (< 0.05). ^b^ Initial components of primary metabolites in R2YE medium at an early stage. ^c^ Initial components of primary metabolites in RSM3 medium at an early stage.
(DOCX)

S1 File The raw data sets of GC-TOF-MS analysis. The raw data sets of GC-TOF-MS were converted to the Net CDF format (*.cdf) using the LECO Chroma TOF software (version 4.44, LECO Corpo). Converted CDF data were preprocessed with the MetAlign software package (http://www.metalign.nl) for peak detection, retention time correction, and alignment. The resulting data, including sample names and peak area information as variables, were exported to an Excel file.
(XLSX)

S2 File The raw data sets of UPLC-Q-TOF-MS. The UPLC-Q-TOF-MS data sets were converted to the Net CDF format using MassLynx DataBridge (version 4.1, USA, Waters Corp.). Converted CDF data were preprocessed with the MetAlign software package (http://www.metalign.nl) for peak detection, retention time correction, and alignment. The resulting data, including sample names and peak area information as variables, were exported to an Excel file.
(XLSX)
