# Supplementary material for: Non-targeted metabolomics unravels a media-dependent prodiginines production pathway in Streptomyces coelicolor A3(2)
Source: PLoS One. 2018 Nov 28;13(11):e0207541. doi: 10.1371/journal.pone.0207541 (PMC6261592; doi:10.1371/journal.pone.0207541)
Supplement: S1 Table — (DOCX) [file pone.0207541.s001.docx]

**S1 Table. Medium composition for *S. coelicolor* A3(2) cultivation.**

|  |  |  |  |  |  |  |  |
| --- | --- | --- | --- | --- | --- | --- | --- |
| **R2YE medium composition** | |  |  |  | **RSM3 medium composition** | |  |
|  |  |  |  |  |  |  |  |
| **Reagents** |  | **Per liter** |  |  | **Reagents** |  | **Per liter** |
|  |  |  |  |  |  |  |  |
| Glucose |  | 10 g |  |  | Galactose |  | 15 g |
| K_2_SO_4_ |  | 0.25 g |  |  | Yeast extract |  | 11 g |
| MgCl_2_ |  | 10.12 g |  |  | MgCl_2_ |  | 5 g |
| Casamino acid |  | 0.1 g |  |  | Agar |  | 3 g |
| Yeast extract |  | 5 g |  |  |  |  |  |
| KH_2_PO_4_ (0.5%, w/v) |  | 10 mL |  |  |  |  |  |
| CaCl_2_ (3.68%, w/v) |  | 80 mL |  |  |  |  |  |
| L-Proline (20%, w/v) |  | 15 mL |  |  |  |  |  |
| TES buffer [pH 7.2] (5.73 %, w/v) |  | 100 mL |  |  |  |  |  |
| Trace elements solution |  | 2 mL |  |  |  |  |  |
|  |  |  |  |  |  |  |  |
| ***Trace elements solution* (1L)** |  |  |  |  |  |  |  |
| ZnCl_2_ |  | 40 mg |  |  |  |  |  |
| FeCl_2_ |  | 200 mg |  |  |  |  |  |
| CuCl_2_ |  | 10 mg |  |  |  |  |  |
| MnCl_2_ |  | 10 mg |  |  |  |  |  |
| Na_2_B_4_O_7_ |  | 10 mg |  |  |  |  |  |
| (NH_4_)_6_Mo_7_O_24_ |  | 10 mg |  |  |  |  |  |
|  |  |  |  |  |  |  |  |
