# Supplementary material for: Non-targeted metabolomics unravels a media-dependent prodiginines production pathway in Streptomyces coelicolor A3(2)
Source: PLoS One. 2018 Nov 28;13(11):e0207541. doi: 10.1371/journal.pone.0207541 (PMC6261592; doi:10.1371/journal.pone.0207541)
Supplement: S2 Table — a Retention time; b Metabolites selected by VIP value > 0.7 based on OPLS-DA (Fig 2B); c It was selected by p-value (< 0.05) based on one-way ANOVA analysis. d Identification. CCD, The Dictionary of Natural Products (version 16:2, 2007, Chapman & Hall, USA); BioCys, Identification of metabolites was carried out using BioCyc Database Collection (https://biocyc.org/); HMDB, Identification of metabolites was carried out using the Human Metabolome Database (HMDB; http://www.hmdb.ca/). (DOCX) [file pone.0207541.s002.docx]

**S2 Table. Secondary metabolites produced by *S. coelicolor* A3(2) grown in R2YE and RSM3 media tentatively identified by time-resolved cultivation analyzed by UPLC-Q-TOF-MS.**

| **No.** | **RT^a^ (min)** | **Tentative Metabolite^b^** | **UPLC-Q-TOF-MS** | | | | | |  | **UHPLC-LTQ-IT-MS/MS** | | **VIP** | **ID^d^** |
| --- | --- | --- | --- | --- | --- | --- | --- | --- | --- | --- | --- | --- | --- |
|  |  |  | **[M+H]^+^** | **[M-H]^-^** | **M.W.** | **M.F.** | **Error (mDa)** | **i-Fit (norm)** |  | **MS^n^ fragment ions** | **UV**  **max (nm)** |  |  |
| ***Prodiginines*** | | | | | | | | | | | |  |  |
| 1 | 7.70 | 4-Keto-2-undecylpyrroline^c^ | 238.2171 | - | 237 | C15H27NO | 0.9 | - |  | 238>196>179 | 223 | 1.94 | Ref[S1], CCD |
| 2 | 8.05 | 23-Hydroxyundecylprodiginine^c^ | 410.2808 | 408.2651 | 409 | C25H35N3O2 | 1.2 | 0.997 |  | 410>392>377>278 | 276 533 | 6.53 | Ref[S2], CCD |
| 3 | 8.18 | Streptorubin B^c^ | 392.2702 | - | 391 | C25H33N3O | -0.6 | 0.334 |  | 392>377>252 | 280 533 | 6.98 | Ref[S3] |
| 4 | 9.02 | Undecylprodigiosin^c^ | 394.2858 | 392.2702 | 393 | C25H35N3O | -3.1 | 0.112 |  | 394>379>251>237 | 270 366 524 | 4.38 | Ref[S3] |
| ***Indoles*** | | | | | | | | | | | | | |
| 5 | 4.09 | Indole-3-acetic acid^c^ | 176.0712 | 174.0555 | 175 | C10H9NO2 | 1.6 | - |  | 176>157>129>117 | 240 257 298 | 2.00 | Ref[S4] |
| 6 | 5.45 | Oxopropaline D^c^ | 271.1103 | 269.0926 | 270 | C15H14N2O3 | -0.1 | - |  | 271>257>239>211>169 | 225 291 | 3.87 | CCD |
| ***Germicidins*** | | | | | | | | | | | | | |
| 7 | 5.02 | Germicidin B^c^ | 183.1021 | 181.0766 | 182 | C10H14O3 | -1.1 | 0.02 |  | 183>155>137>109 | 207 288 | 8.80 | Ref[S5], BioCyc |
| 8 | 5.54 | Germicidin A^c^ | 197.1178 | 195.1021 | 196 | C11H16O3 | 1 | - |  | 197>168>151>123>97 | 204 291 | 6.45 | Ref[S6] |
| ***Other antibiotics*** | | | | | | | | | | | | | |
| 9 | 4.03 | Phaeochromycin G^c^ | 217.0854 | - | 216 | C13H12O3 | 0.1 | - |  | 217>174>162 | 240 291 | 8.06 | Ref[S7] |
| 10 | 5.95 | Antibiotic KF 77AG6^c^ | 366.1777 | - | 365 | C16H23N5O5 | -2 | 1.192 |  | 366>348>321>226 | 218 284 301 | 12.46 | CCD |
| 11 | 6.23 | Violapyrone J^c^ | 211.1334 | - | 210 | C12H18O3 | -0.6 | 0.125 |  | 211>183>155>138 | 220 275(sh) | 5.94 | Ref[S8] |
| ***Diketopiperazines*** | | | | | | | | | | | | | |
| 12 | 3.94 | Gancidin W^c^ | 211.1370 | - | 210 | C11H18N2O2 | -0.4 | - |  | 211>183>154 | 268 | 8.09 | HMDB, CCD |
| 13 | 4.28 | Tryptophandehydrobutyrine diketopiperazine^c^ | 284.1399 | - | 283 | C16H17N3O2 | 1 | 0.008 |  | 284>267>130 | 250 282 | 8.75 | CCD |
| 14 | 4.95 | Cyclo(leucylphenylalanyl)^c^ | 261.1603 | - | 260 | C15H20N2O2 | 1.3 | 0.004 |  | 261>133>188>120 | 225 290 | 8.22 | Ref[S9], CCD |
| 15 | 5.99 | Cyclo(phenylalanyl-N-methyltryptophyl)^c^ | 348.1712 | - | 347 | C21H21N3O2 | 1.7 | 0.971 |  | 348>320>303>264 | 219 270(sh) | 5.40 | CCD |

^a^ Retention time; ^b^ Metabolites selected by VIP value > 0.7 based on OPLS-DA (Fig 1b); ^c^ It was selected by p-value (< 0.05) based on one-way ANOVA analysis. ^d^ Identification. CCD, *The Dictionary of Natural Products* (version 16:2, 2007, Chapman & Hall, USA); BioCys, Identification of metabolites was carried out using BioCyc Database Collection (https://biocyc.org/); HMDB, Identification of metabolites was carried out using the Human Metabolome Database (HMDB; http://www.hmdb.ca/).

**Supplementary references**

1. Cerdeno AM, Bibb MJ, Challis GL. Analysis of the prodiginine biosynthesis gene cluster of *Streptomyces coelicolor* A3(2): new mechanisms for chain initiation and termination in modular multienzymes. Chem Biol. 2001; 8(8):817-29. doi: 10.1016/S1074-5521(01)00054-0
2. Salem SM, Kancharla P, Florova G, Gupta S, Lu W, Reynolds KA. Elucidation of final steps of the marineosins biosynthetic pathway through identification and characterization of the corresponding gene cluster. J Am Chem Soc. 2014; 136(12): 4565–4574. doi: 10.1021/ja411544w
3. Meschke H, Walter S, Schrempf H. Characterization and localization of prodiginines from *Streptomyces lividans* suppressing *Verticillium dahliae* in the absence or presence of *Arabidopsis thaliana*. Environ Microbiol. 2012; 14(4):940-52. 10.1111/j.1462-2920.2011.02665.x
4. Matsuda F, Miyazawa H, Wakasa K, Miyagawa H. Quantification of indole-3-acetic acid and amino acid conjugates in rice by liquid chromatography-electrospray ionization-tandem mass spectrometry. Biosci Biotechnol Biochem. 2005; 69(4):778-83. doi: 10.1271/bbb.69.778
5. Petersen F, Zahner H, Metzger JW, Freund S, Hummel RP. Germicidin, an autoregulative germination inhibitor of *Streptomyces viridochromogenes* NRRL B-1551. J Antibiot (Tokyo). 1993; 46(7): 1126-1138. doi: 10.7164/antibiotics.46.1126
6. Cihak M, Kamenik Z, Smidova K, Bergman N, Benada O, Kofronova O, et al. Secondary metabolites produced during the germination of *Streptomyces coelicolor*. Front Microbiol. 2017; 8:2495. doi: 10.3389/fmicb.2017.02495
7. Li J, Lu CH, Zhao BB, Zheng ZH, Shen YM. Phaeochromycins F-H, three new polyketide metabolites from *Streptomyces sp*. DSS-18. Beilstein J Org Chem. 2008; 4:46. doi: 10.3762/bjoc.4.46
8. Leutou AS, Yang I, Seong CN, Ko J, Nam S. Violapyrone J, α-pyrone derivative from a marine-derived actinomycetes, *Streptomyces* sp. Nat Prod Sci. 2015; 21(4):248. doi: 10.20307/nps.2015.21.4.248
9. Gondry M, Sauguet L, Belin P, Thai R, Amouroux R, Tellier C, et al. Cyclodipeptide synthases are a family of tRNA-dependent peptide bond-forming enzymes. Nat Chem Biol. 2009; 5(6):414-20. doi:10.1038/nchembio.175
