# Supplementary material for: Non-targeted metabolomics unravels a media-dependent prodiginines production pathway in Streptomyces coelicolor A3(2)
Source: PLoS One. 2018 Nov 28;13(11):e0207541. doi: 10.1371/journal.pone.0207541 (PMC6261592; doi:10.1371/journal.pone.0207541)
Supplement: S3 Table — a Retention time; b Metabolites selected by VIP value > 0.7 based on PLS-DA (S1C Fig); c It was selected by p-value (< 0.05) based on one-way ANOVA analysis; d Identification. MS, mass spectrum was confirmed with the National Institutes of Standards and Technology (NIST) database and in-house libraries; STD, mass spectrum was consistent with that of the standard compounds; TMS, trimethylsilyl. (DOCX) [file pone.0207541.s003.docx]

**S3 Table. Primary metabolites produced by *S. coelicolor* A3(2) grown in R2YE medium tentatively identified by time-resolved cultivation analyzed by GC-TOF-MS.**

| **No.** |  | | **RT^a^(min)** | **Tentative Metabolite^b^** | **Mass** | **MS Fragment** | **VIP1** | **VIP2** | **TMS** | **ID^d^** |
| --- | --- | --- | --- | --- | --- | --- | --- | --- | --- | --- |
|  | | ***Amino acids*** | | | | | | | | |
| 1 |  | | 5.63 | L-Alanine^c^ | 233 | 116 45 117 75 59 | 1.27 | 0.93 | 2 | STD |
| 2 |  | | 6.78 | L-Valine^c^ | 261 | 144 45 218 145 100 | 1.35 | 1.06 | 2 | STD |
| 3 |  | | 7.32 | L-Leucine^c^ | 275 | 158 45 102 159 74 | 0.98 | 0.73 | 2 | STD |
| 4 |  | | 7.54 | L-Isoleucine^c^ | 275 | 158 45 74 75 218 | 1.15 | 0.86 | 2 | STD |
| 5 |  | | 7.59 | L-Proline^c^ | 259 | 142 45 66 74 59 | 0.37 | 1.40 | 2 | STD |
| 6 |  | | 7.67 | Glycine^c^ | 291 | 86 174 100 45 59 | 1.52 | 1.09 | 3 | STD |
| 7 |  | | 8.40 | L-Threonine^c^ | 335 | 57 117 101 219 45 | 1.59 | 1.36 | 3 | STD |
| 8 |  | | 9.52 | Aspartic acid^c^ | 349 | 75 45 61 129 74 | 1.97 | 1.68 | 3 | STD |
| 9 |  | | 9.53 | L-Methionine^c^ | 293 | 176 128 61 45 57 | 1.58 | 1.25 | 2 | STD |
| 10 |  | | 9.58 | Pidolic acid | 273 | 156 45 157 74 59 | 0.00 | 1.15 | 1 | STD |
| 11 |  | | 9.61 | GABA | 319 | 174 86 45 59 175 | 0.85 | 0.71 | 3 | STD |
| 12 |  | | 10.30 | Glutamic acid^c^ | 363 | 246 75 128 45 84 | 1.78 | 1.27 | 3 | STD |
| 13 |  | | 10.41 | Phenylalanine^c^ | 309 | 218 192 100 45 74 | 1.28 | 0.98 | 2 | STD |
| 14 |  | | 11.80 | L-Ornithine^c^ | 420 | 142 174 45 74 89 | 1.16 | 0.83 | 4 | MS |
| 15 |  | | 12.52 | L-Lysine^c^ | 434 | 174 156 128 86 59 | 1.78 | 1.41 | 4 | STD |
| 16 |  | | 12.65 | L-Tyrosine^c^ | 397 | 218 100 219 45 74 | 1.80 | 1.37 | 3 | STD |
| 17 |  | | 14.43 | L-Tryptophan^c^ | 420 | 202 203 45 74 291 | 1.73 | 1.37 | 3 | STD |
|  | | ***Sugars and Sugar alcohols*** | | | | | | | | |
| 18 |  | | 7.34 | Glycerol^c^ | 308 | 117 103 45 205 75 | 1.50 | 1.45 | 3 | STD |
| 19 |  | | 7.88 | Glyceric acid^c^ | 322 | 189 45 103 102 133 | 1.58 | 1.14 | 3 | MS |
| 20 |  | | 9.97 | 2-Deoxy-D-ribose^c^ | 379 | 142 103 45 174 75 | 1.30 | 1.38 | 3 | MS |
| 21 |  | | 10.63 | 2-Keto-gluconic acid | 583 | 204 103 117 45 205 | 0.38 | 1.00 | 5 | MS |
| 22 |  | | 11.04 | Xylitol^c^ | 512 | 129 45 204 103 217 | 1.63 | 1.57 | 5 | STD |
| 23 |  | | 12.25 | Tagatose^c^ | 569 | 103 217 307 218 104 | 1.65 | 1.72 | 5 | STD |
| 24 |  | | 12.46 | D-Glucose^c^ | 569 | 205 160 103 319 217 | 1.73 | 1.53 | 5 | STD |
| 25 |  | | 13.69 | *myo*-Inositol^c^ | 612 | 217 191 305 103 45 | 1.54 | 1.23 | 6 | STD |
|  | | ***Fatty acids*** | | | | | | | | |
| 26 |  | | 6.27 | 2-Hydroxy-2-methylbutyric acid | 262 | 145 75 45 74 146 | 1.01 | 1.00 | 2 | MS |
| 27 |  | | 8.48 | Glutaric acid | 276 | 55 75 45 158 97 | 1.04 | 0.88 | 2 | MS |
| 28 |  | | 8.60 | 3-Deoxytetronic acid | 336 | 103 219 45 129 55 | 1.24 | 1.04 | 3 | MS |
| 29 |  | | 10.38 | Valeric acid, 5-amino- | 333 | 174 82 86 45 175 | 0.95 | 0.94 | 3 | MS |
| 30 |  | | 13.50 | Oleanitrile | 263 | 55 69 56 122 83 | 0.37 | 0.97 | 0 | MS |
| 31 |  | | 14.26 | Elaidic acid | 326 | 75 117 55 129 67 | 1.24 | 0.89 | 1 | STD |
| 32 |  | | 14.39 | Stearic acid | 356 | 117 75 132 129 55 | 1.08 | 0.77 | 1 | STD |
| 33 |  | | 15.39 | Oleamide | 353 | 75 131 144 116 128 | 0.80 | 1.08 | 1 | STD |
| 34 |  | | 16.28 | 1-Monopalmitin | 474 | 57 55 75 129 71 | 0.95 | 1.09 | 2 | MS |
|  | | ***Organic acid*** | | | | | | | | |
| 35 |  | | 5.20 | Lactic acid | 234 | 117 45 66 75 74 | 0.90 | 1.15 | 2 | STD |
| 36 |  | | 5.34 | Glycolic acid^c^ | 220 | 66 45 177 148 74 | 1.04 | 1.49 | 2 | MS |
| 37 |  | | 6.98 | Urea^c^ | 204 | 171 189 45 66 148 | 1.43 | 1.20 | 2 | MS |
| 38 |  | | 7.79 | 2,3-Dihydroxy-2-methylpropanoic acid | 336 | 75 219 129 45 131 | 1.05 | 1.12 | 3 | MS |
| 39 |  | | 10.28 | Anthranilic acid^c^ | 281 | 266 45 267 75 118 | 1.56 | 1.36 | 2 | MS |
|  | | ***Alcohols*** | | | | | | | | |
| 40 |  | | 4.59 | Propylene glycol^c^ | 220 | 117 66 75 74 59 | 1.34 | 1.55 | 2 | MS |
| 41 |  | | 5.84 | 2-Butene-1,4-diol | 232 | 129 66 45 75 69 | 0.87 | 0.62 | 2 | MS |
| 42 |  | | 9.45 | Erythritol | 410 | 103 217 117 45 205 | 1.08 | 0.82 | 4 | MS |
|  | | ***Others*** | | | | | | | | |
| 43 |  | | 5.76 | Hydroxylamine | 249 | 133 146 119 59 86 | 0.78 | 0.63 | 3 | MS |
| 44 |  | | 7.36 | Phosphoric acid | 314 | 45 299 133 74 314 | 0.66 | 1.18 | 3 | STD |
| 45 |  | | 7.96 | Uracil | 256 | 99 241 45 113 255 | 1.23 | 0.88 | 2 | MS |
| 46 |  | | 13.71 | Uric acid | 456 | 45 74 441 456 100 | 0.33 | 1.18 | 4 | MS |

^a^ Retention time; ^b^ Metabolites selected by VIP value > 0.7 based on PLS-DA (S1c Fig); ^c^ It was selected by p-value (< 0.05) based on one-way ANOVA analysis; ^d^ Identification. MS, mass spectrum was confirmed with the National Institutes of Standards and Technology (NIST) database and in-house libraries; STD, mass spectrum was consistent with that of the standard compounds; TMS, trimethylsilyl.
