# Supplementary material for: Non-targeted metabolomics unravels a media-dependent prodiginines production pathway in Streptomyces coelicolor A3(2)
Source: PLoS One. 2018 Nov 28;13(11):e0207541. doi: 10.1371/journal.pone.0207541 (PMC6261592; doi:10.1371/journal.pone.0207541)

**S1 Fig. PCA score plots with QC of primary (A) and secondary metabolites (B) produced by *S. coelicolor* A3(2) grown in R2YE and RSM3 media, and PLS-DA score plots of primary metabolites (C) and secondary metabolites (D) produced by *S. coelicolor* A3(2) grown in R2YE medium.** The analysis was performed with GC-TOF-MS and UPLC-Q-TOF-MS. Data were scaled to internal standards using methyl nonadecanoate (for GC-TOF-MS analysis) and adenosine 5′-monophosphate monohydrate (for UPLC-Q-TOF-MS analysis). (Star, quality control; square, *S. coelicolor* A3(2) in R2YE medium; triangle, *S. coelicolor* A3(2) in RSM3; yellow, 48 h; orange, 72 h; red, 96 h; purple, 120 h; blue, 144 h; green, 168 h).


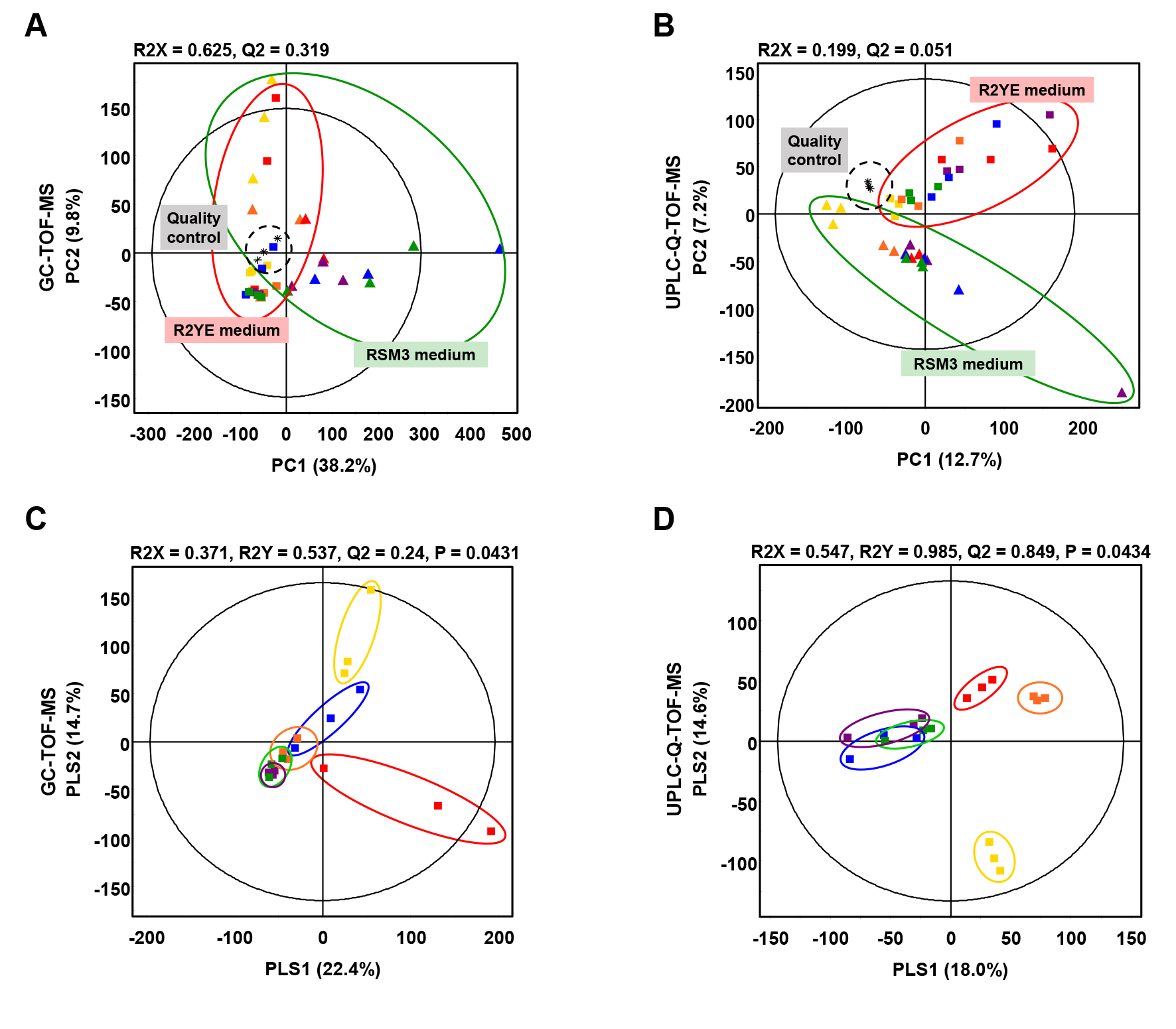

Supplement: S1 Fig — PCA score plots with QC of primary (A) and secondary metabolites (B) produced by S. coelicolor A3(2) grown in R2YE and RSM3 media, and PLS-DA score plots of primary metabolites (C) and secondary metabolites (D) produced by S. coelicolor A3(2) grown in R2YE medium. The analysis was performed with GC-TOF-MS and UPLC-Q-TOF-MS. Data were scaled to internal standards using methyl nonadecanoate (for GC-TOF-MS analysis) and adenosine 5′-monophosphate monohydrate (for UPLC-Q-TOF-MS analysis). (Star, quality control; square, S. coelicolor A3(2) in R2YE medium; triangle, S. coelicolor A3(2) in RSM3; yellow, 48 h; orange, 72 h; red, 96 h; purple, 120 h; blue, 144 h; green, 168 h). (DOCX) [file pone.0207541.s005.docx]
