# Supplementary material for: Non-targeted metabolomics unravels a media-dependent prodiginines production pathway in Streptomyces coelicolor A3(2)
Source: PLoS One. 2018 Nov 28;13(11):e0207541. doi: 10.1371/journal.pone.0207541 (PMC6261592; doi:10.1371/journal.pone.0207541)

**S2 Fig. Metabolites in R2YE (A) and RSM3 (B) analyzed by GC-TOF-MS.** Metabolites were tentatively identified by comparison with mass fragment patterns, retention time, and mass spectrum of analysis data for standard compounds under the same conditions and commercial databases, such as the NIST Library and Wiley 8. Metabolite numbers are as follows: 1. L-alanine, 2. L-valine, 3. L-leucine, 4. L-isoleucine, 5. L-proline, 6. glycine, 7. serine, 8. L-threonine, 9. aspartic acid, 10. L-methionine, 11. pidolic acid, 12. GABA, 13. glutamic acid, 14. phenylalanine, 15. L-ornithine, 16. L-lysine, 17. L-tyrosine, 18. L-tryptophan, 19. glycerol, 20. glyceric acid, 21. threonic acid, 22. 2-deoxy-D-ribose, 23. 2-keto-gluconic acid, 24. xylose, 25. arabinose, 26. xylitol, 27. tagatose, 28. D-galactose, 29. D-glucose, 30. D-gluconic acid, 31. myo-inositol, 32. 2-hydroxy-2-methylbutyric acid, 33. 4-hydroxybutanoic acid, 34. glutaric acid, 35. 3-deoxytetronic acid, 36. valeric acid, 5-amino-, 37. oleanitrile, 38. elaidic acid, 39. stearic acid, 40. oleamide, 41. 1-monopalmitin, 42. lactic acid, 43. glycolic acid, 44. urea, 45. benzoic acid, 46. 2,3-dihydroxy-2-methylpropanoic acid, 47. anthranilic acid, 48. propylene glycol, 49. 2,3-butanediol, 50. 2-butene-1,4-diol, 51. carbitol, 52. erythritol, 53. uracil, 54. cytosine, 55. 2-pyrrolidinone, 56. hydroxylamine, 57. phosphoric acid, and 58. uric acid.
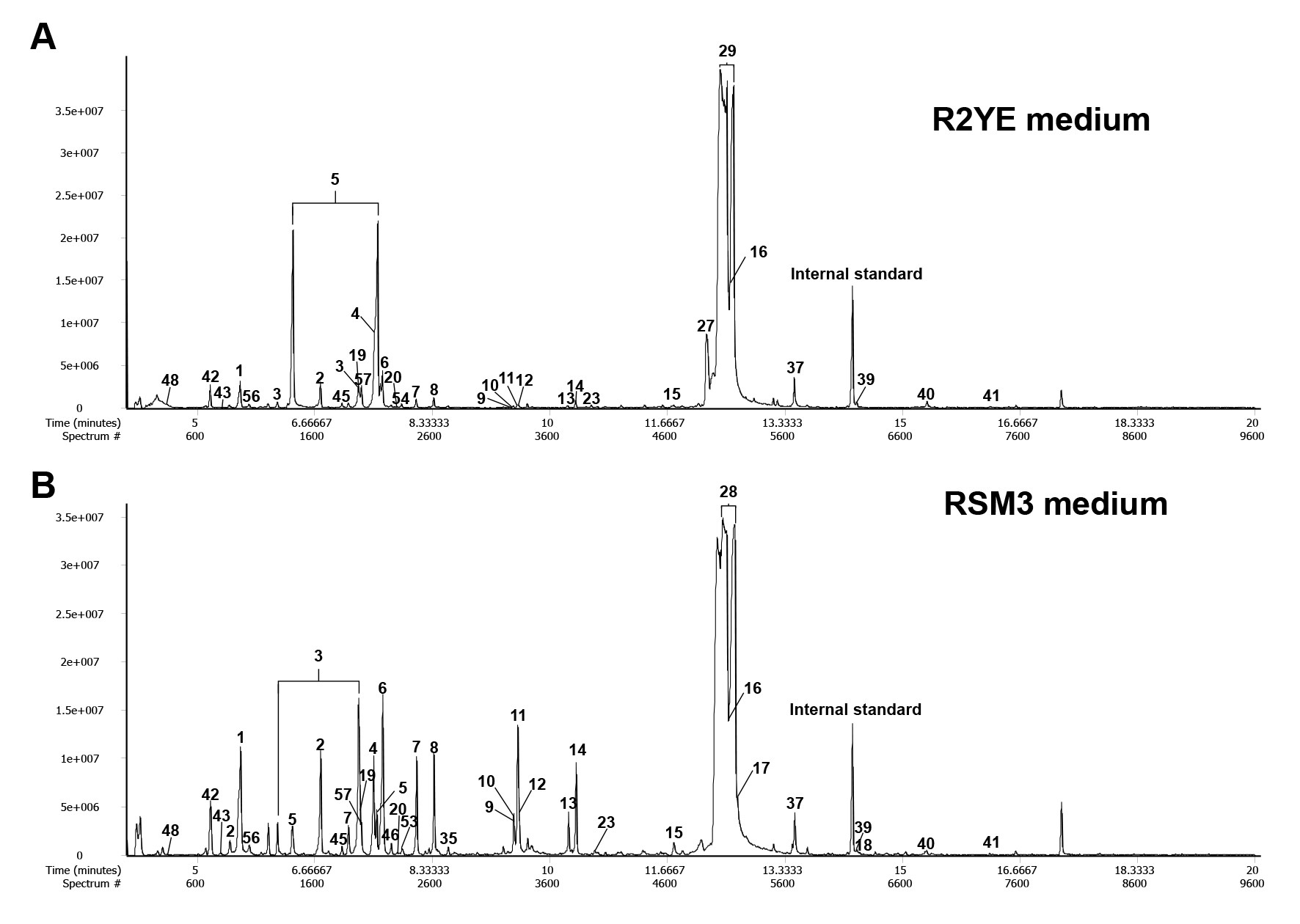

Supplement: S2 Fig — Metabolites in R2YE (A) and RSM3 (B) analyzed by GC-TOF-MS. Metabolites were tentatively identified by comparison with mass fragment patterns, retention time, and mass spectrum of analysis data for standard compounds under the same conditions and commercial databases, such as the NIST Library and Wiley 8. Metabolite numbers are as follows: 1. L-alanine, 2. L-valine, 3. L-leucine, 4. L-isoleucine, 5. L-proline, 6. glycine, 7. serine, 8. L-threonine, 9. aspartic acid, 10. L-methionine, 11. pidolic acid, 12. GABA, 13. glutamic acid, 14. phenylalanine, 15. L-ornithine, 16. L-lysine, 17. L-tyrosine, 18. L-tryptophan, 19. glycerol, 20. glyceric acid, 21. threonic acid, 22. 2-deoxy-D-ribose, 23. 2-keto-gluconic acid, 24. xylose, 25. arabinose, 26. xylitol, 27. tagatose, 28. D-galactose, 29. D-glucose, 30. D-gluconic acid, 31. myo-inositol, 32. 2-hydroxy-2-methylbutyric acid, 33. 4-hydroxybutanoic acid, 34. glutaric acid, 35. 3-deoxytetronic acid, 36. valeric acid, 5-amino-, 37. oleanitrile, 38. elaidic acid, 39. stearic acid, 40. oleamide, 41. 1-monopalmitin, 42. lactic acid, 43. glycolic acid, 44. urea, 45. benzoic acid, 46. 2,3-dihydroxy-2-methylpropanoic acid, 47. anthranilic acid, 48. propylene glycol, 49. 2,3-butanediol, 50. 2-butene-1,4-diol, 51. carbitol, 52. erythritol, 53. uracil, 54. cytosine, 55. 2-pyrrolidinone, 56. hydroxylamine, 57. phosphoric acid, and 58. uric acid. (DOCX) [file pone.0207541.s006.docx]
