# Supplementary material for: Non-targeted metabolomics unravels a media-dependent prodiginines production pathway in Streptomyces coelicolor A3(2)
Source: PLoS One. 2018 Nov 28;13(11):e0207541. doi: 10.1371/journal.pone.0207541 (PMC6261592; doi:10.1371/journal.pone.0207541)

**S3 Fig. Heat map representations of the relative content of significantly discriminant primary metabolites produced by *S. coelicolor* A3(2) cultivated in R2YE and RSM3 media analyzed by GC-TOF-MS**. Significantly discriminant metabolites were selected by VIP values > 0.7. Fold change was normalized to an average of all values and is shown as blue (0.0) to red (2.0). ^a^ Selection by *p*-value (< 0.05). ^b^ Initial components of primary metabolites in R2YE medium at an early stage. ^c^ Initial components of primary metabolites in RSM3 medium at an early stage.


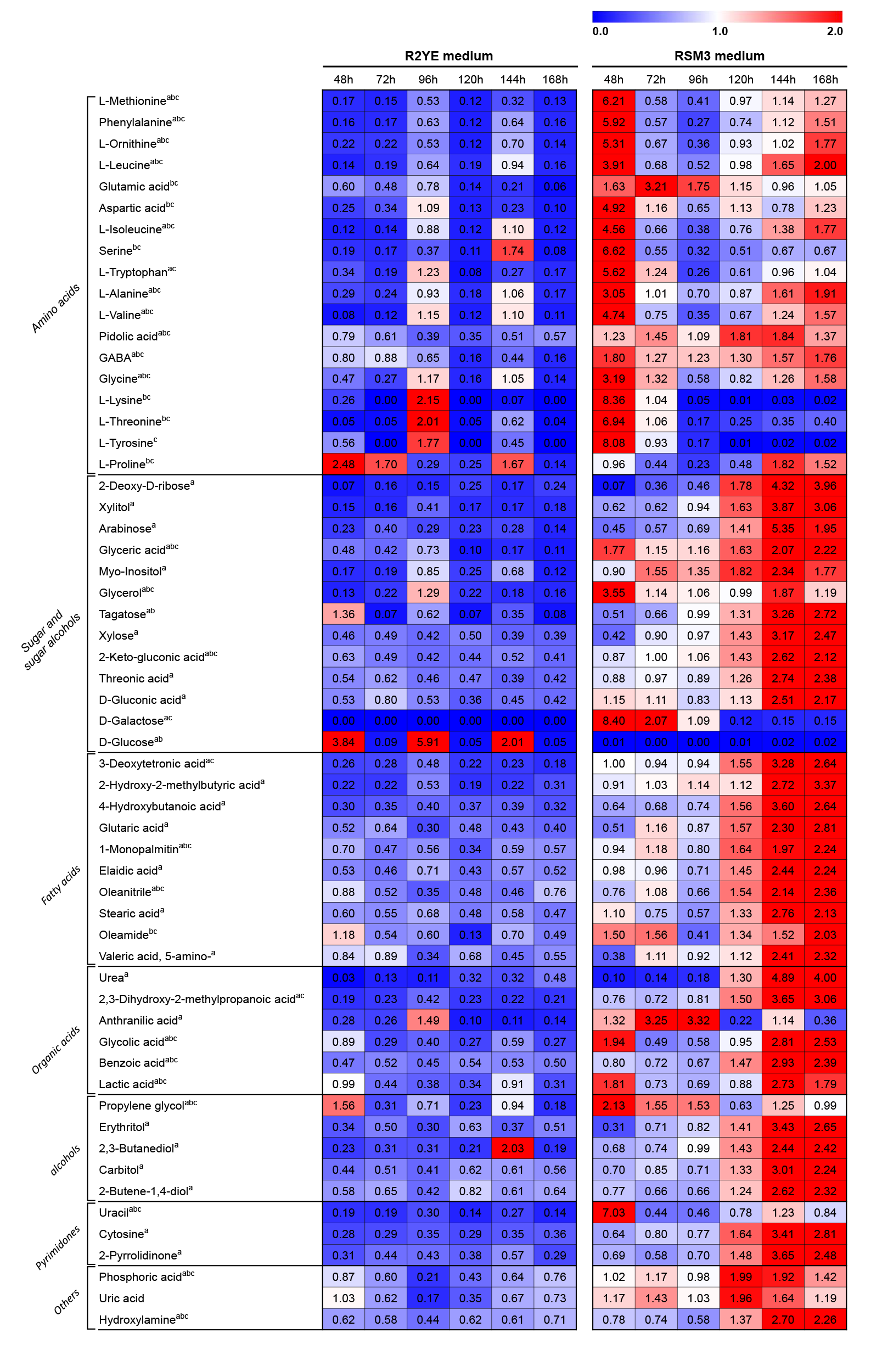

Supplement: S3 Fig — Significantly discriminant metabolites were selected by VIP values > 0.7. Fold change was normalized to an average of all values and is shown as blue (0.0) to red (2.0). a Selection by p-value (< 0.05). b Initial components of primary metabolites in R2YE medium at an early stage. c Initial components of primary metabolites in RSM3 medium at an early stage. (DOCX) [file pone.0207541.s007.docx]
